# Anatomy, Physiology, and Disorders of the Spectacle, Subspectacular Space, and Its Lacrimal Drainage System in Squamates

**DOI:** 10.3390/ani13061108

**Published:** 2023-03-21

**Authors:** Tom Hellebuyck, Ferran Solanes Vilanova

**Affiliations:** Department of Pathobiology, Pharmacology and Zoological Medicine, Faculty of Veterinary Medicine, Ghent University, Salisburylaan 133, B-9820 Merelbeke, Belgium

**Keywords:** lizards, pseudobupthalmos, snakes, spectacle, subspectacular abscess

## Abstract

**Simple Summary:**

Various snake and lizard species have transparent and fused eyelids that make up a spectacle. From an evolutionary point of view, it is assumed that ancestral snakes developed this structure to protect the eye as an adaptation to their burrowing, underground existence. Between the spectacle and the eye lies a narrow subspectacular space that is filled with a tear-like fluid that drains to the roof of the mouth via the lacrimal duct. Although disorders of the eye are relatively uncommon in squamates with a spectacle, disorders of the spectacle, the subspectacular space, and its drainage system are frequently encountered in veterinary practice. As the spectacle is an integral part of the skin it is renewed with each shedding cycle. Retention of the old layers of the spectacle may occur because of infectious as well as non-infectious causes. Besides inflammation and trauma of the spectacle, overfilling due to blockage of the drainage system (pseudobuphthalmos) as well as bacterial and parasitic infection of the subspectacular space (subspectacular infection) are undoubtedly the most frequently observed disorders in snakes and spectacled lizards. An adequate diagnostic approach often allows the establishment of a successful treatment for these conditions.

**Abstract:**

Various squamate species have completely fused eyelids that make up a transparent spectacle. The spectacle is a continuation of the integument that is renewed with each shedding cycle and creates a narrow subspectacular or corneospectacular space that is filled with lacrimal fluid. The latter is considered as the analogue of the conjunctival sac in other vertebrates. Almost all reptiles that have a spectacle lack a nictitating membrane, bursalis muscle, and lacrimal glands. The lacrimal fluid in the subspectacular space is secreted by the Harderian gland. The features of the spectacle and its lacrimal drainage system are an illustration of the enormous variation of the morphological adaptations that are seen in reptiles and one of the most distinguishable traits of snakes and most gecko species. Whereas ocular disease in squamates with a spectacle is infrequently seen in practice, disorders of the spectacle and the subspectacular space are commonly encountered. In order to apply an adequate diagnostic and therapeutic approach for these conditions, a sound knowledge and understanding of the anatomical and physiological peculiarities of the spectacle, subspectacular space, and lacrimal drainage system are fundamental.

## 1. Introduction

**Figure 1:**
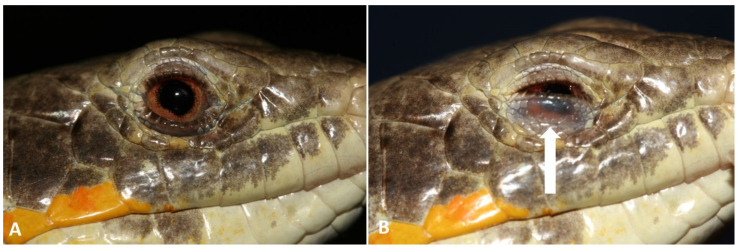
(**A**) Schneider’s skink (*Eumeces schneiderii*); this species has a window in the lower eyelid (arrow) made up by multiple transparent scales (**B**).

It is generally accepted that moveable eyelids are the primitive condition for squamata [1,2,3,4]. In several squamate lineages, however, the eyelids may show a varying degree of modification ranging from thinning of the dermal layers to the presence of a single transparent scale or multiple transparent scales creating a window in the lower eyelid (Figure 1) or covering the eye. In various taxa, moveable eyelids have been completely replaced by a truly transparent and rigid spectacle (Table 1) [2,3,4,5,6]. The reptilian spectacle is considered the most sophisticated type of spectacle (tertiary spectacle) that is posteriorly lined with conjunctival epithelium bordering a narrow subspectacular or corneospectacular space filled with a fluid analogous to the tear film of lidded vertebrates allowing the eye to rotate freely [2,3,6].

**Table 1:** Overview of squamate lineages that show a varying degree of modification of the eyelids or possess a truly transparent and rigid spectacle.

Clade/Superfamily	Family	Comments
Amphisbaenia (Worm lizards)	Amphisbaenidae Bipedidae Blanidae Cadeidae Rhineuridae Trogonophidae	Reduced eyes. Moveable eyelids have been replaced by a transparent ocular scale that covers the eye (Figure 2A,B). Sometimes the ocular scale is not thinner than the skin and pigmentation might be present [2,6].
Dibamidae (Blind lizards)	Dibamidae	Reduced eyes. Moveable eyelids have been replaced by transparent scales that cover the eye [4,6].
Gekkota	Pygopodidae	Only the clade of the eublepharid geckos (*Eublepharidae*) has fully moveable eyelids. A true transparent and rigid spectacle is present in all other Gekkota [1,5,6,7,8,9].
	Diplodactylidae	
	Gekkonidae	
	Phyllodactylidae	
	Sphaerodactylidae	
Scincomorpha	Scincidae (skinks)	Many species belonging to various genera have a transparent window in the lower eyelid made up by multiple transparent scales (Figure 1). Genera *Leiolopisma* and *Trachylepsis* (formerly *Mabuya*): single large transparent scale in the lower eyelid large enough to cover the cornea [6,10,11,12]. Genera *Ablepharus* (Figure 2C), *Morethia*, *Proablepharus*, and *Cryptoblepharus:* true spectacle [5,12]. Genus *Ablepharus*: only genus in which lacrimal glands are present [4,5,6].
	Xantusiidae (Night lizards)	True spectacle (Figure 2D) [2,4,13,14].
Lacteroidea	Gymnophtalmidae (Spectacled lizards, microteiids)	Most species: moveable upper eyelids and immobile lower eyelids with a transparent window [3,4,14,15,16,17]. Monophyletic tribe Gymnophtalmini: true spectacle [15,18,19].
	Lacertidea	Genus *Eremias*: simplest eyelid modification involving a thinning of the layers of multiple scales creating an ‘eyelid window’. Genus *Ophisops:* true spectacle.
Typhlopoidea (Scolecophidia, Blind snakes)		Reduced eyes. Transparent ocular scale rather than a spectacle. The scale extends well beyond the margins of the globe, in some cases to the mouth (oculolabial scale) (Figure 2E,F) [2,3,20,21].
All other snakes than Blind snakes (Alethinophidia)		True spectacle [2,3,20,21].

**Figure 3:**
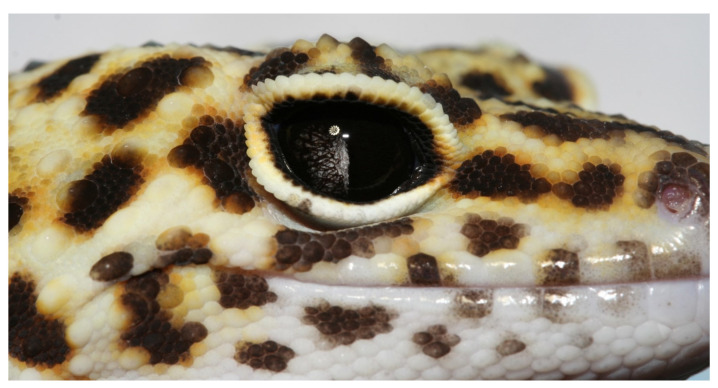
Members of the eublepharid clade, such as this leopard gecko (*Eublepharis macularius*), are the only Gekkota that possess fully moveable eyelids.

**Figure 4:**
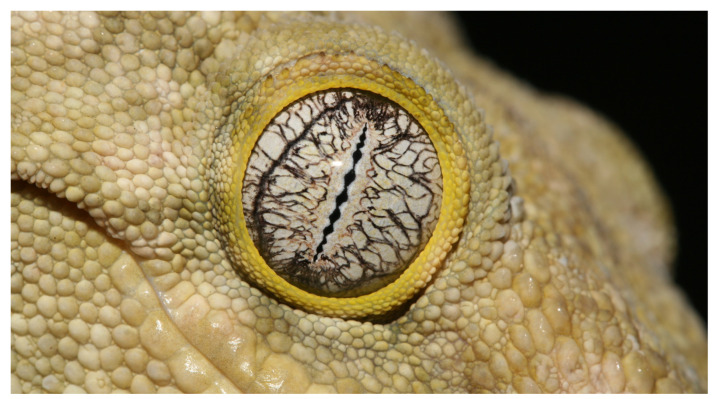
The spectacle in a New Caledonian giant gecko (*Rhacodactylus laechianus*) is bordered by a extra-brillar ring that resembles eyelids but is a separate, fixed structure.

Outside of the squamata, modification of the eyelids is only seen in a few turtle species (e.g., the Indian flapshell turtle (*Lissemys punctata*) and the eastern long-necked turtle (*Chelodina longicollis*)) that have a single large transparent scale in the lower eyelid large enough to cover the cornea [13]. Except for some burrowing snakes (Scolecophidia: Typhlopoidea, blind snakes) that possess an ocular or oculolabial scale and species of the eublepharid geckos that possess fully moveable eyelids (Figure 3), snakes and geckos (Figure 4) are the only taxa where the spectacle occurs in all species regardless of habitat and ecology (Table 1) [2,3,20,21,22,23]. The phylogenetic position of eublepharid geckos suggests that having functional eyelids is a re-aquisited feature of more primitive forms, although spectacles may have also evolved independently in non-eublepharid geckos and their sister taxa [7,8,9,22].

Although we might never fully understand what prompted the ancestors of snakes to develop a spectacle, it is generally accepted that snakes evolved from burrowing lizards that lived underground and the reduced eyes and spectacle are adaptations to this burrowing existence to primarily protect the eye. It would not have been until snakes re-emerged from their underground existence that they re-developed functional eyes that were initially adapted to nocturnal conditions [2,3,6,23,24].

Although it was first hypothesized that the spectacle evolved from a modification of the nictitating membrane [13], studies in natricid and viperid snakes demonstrated that the spectacle is derived from the concentric fusion of the mesenchymal tissues that form the eyelids at the level of the pupil during the embryonic stage. The fused eyelids become transparent and are voided of all glands and muscles [6,10,24,25,26]. The spectacle is well developed at the time of birth in ovoviviparous and oviparous snakes, but in some species, fusion of the eyelids and formation of the spectacle occurs post-oviposition [3,5]. Most species belonging to the Gymnophthalmidae family, also referred to as the spectacled lizards or microteiids, have moveable upper eyelids and immobile lower eyelids with a transparent window [4]. Within this family, the members of the monophyletic tribe Gymnophtalmini have a true spectacle and embryonic fusion of the eyelids takes place above the pupil region [3,4,15,16,17,18,19]. It remains uncertain, however, if functional eyelids can be either considered as a primitive condition or as a re-acquisition of the character in *Tretrioscincus* species belonging to the single genus of the tribe where functional eyelids are present [4]. The spectacle of other saurian species and the ocular scale of amphisbaenians and blind snakes are also considered to be formed by fusion of the eyelids, but it is unknown if the embryonic development pattern equals that of snakes or Gymnophthalmini [1,4]. In amphisbaenians, the thickness of the ocular scale and its degree of pigmentation is variable, suggesting that the emphasis placed on vision varies significantly among these fossorial squamates [2].

## 2. The Structure and Function of the Spectacle in Squamates

**Figure 5:**
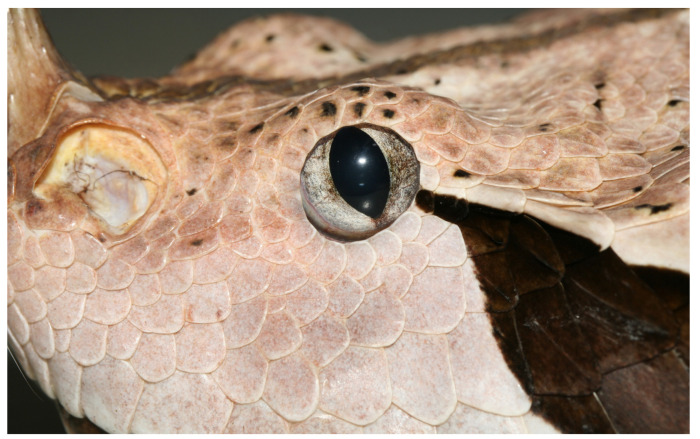
The transition zone between the periocular scales and the spectacle can be clearly distinguished in this West African Gaboon viper (*Bitis gabonica* rhinoceros).

The periocular scales form a discrete rim that continues in a transition zone of dermis that gradually becomes thinner and connects with the spectacle (Figure 5) [27,28,29].

Histologically, the spectacle consists of three layers and has been best studied in snakes [30,31]. The outer epithelial layer (3.5–10.5 μm) has one (germinal layer) or more layers of basal cells and is covered by keratin made up of 2 to 4 alternating layers of alpha and beta keratin depending on the stage of the shedding cycle. Beta keratin composition seems to differ between species and bears a relationship with taxonomy, suggesting that optical transparency is not restricted to a few isoforms [32]. The inner epithelial layer (1.6–3.6 μm) is a single germinal layer that is considered as the continuation of the palpebral conjunctiva and is separated from the underlying stroma layer by a basement membrane. The free border of the inner epithelium that delineates the lacrimal-fluid-filled subspectacular space is considered as the homologue of the corneal endothelium [31]. The stroma (9–132 μm) contains parallel layers of evenly spaced lamellar of collagen fibrils with an alternating course, similar to what is seen in the vertebrate cornea. In contrast to the cornea, however, the stroma layer contains blood vessels, fibroblasts, and nerve fibers [2,31].

In snakes, spectacular thickness seems to reflect evolutionary adaptation and development to their natural habitat and ranges from 74 to 244 µm. The thinnest spectacles are found in arboreal and terrestrial species, whereas the thickest occur in aquatic and fossorial or burrowing species. In general, colubrids have thinner spectacles than boas and pythons. The thinnest spectacles are found in viperids and the thickest in pipe snakes [33].

In addition to the protective role of the spectacle, it also constitutes the main refractive ocular surface (refractive index n ≥ 1.5) and plays a crucial role in the quality of vision [2,31,32]. The refractory potential of the spectacle together with the lacrimal fluid in the subspectacular space equals that of the lens in species that do not possess a spectacle and renders the role of the cornea in refraction irrelevant [34]. The spectral transmittance has been studied in snakes as well as lizards and a wide range of wavelengths from red light to ultraviolet (UV) (280–315 nm) are transmitted; however, the spectacle also acts as an optical filter that reduces the passage of UV irradiation with short wavelengths in diurnal species [35,36].

Although the spectacle is optically transparent, similar to other parts of the integument it is well vascularized [37]. As spectacled reptiles are nearly the only vertebrates that possess non-retinal blood vessels in the visual fields, adaptations to minimize loss of clarity in the optical transmissive regions of the eye are necessary. In addition to transparent blood vessel walls and a specific spatial layout and density in the spectral regions that serve the foveal and binocular visual fields, it has been shown that these vessels undergo cycles of dilation and constriction to impact visual clarity as minimally as possible in snakes [2,37]. During activity or whenever a snake is visually stimulated, spectacle vessels remain constricted for longer periods than during a resting phase. During the renewal phase of the shedding cycle, spectacle vessels remain dilated and blood flow remains strong and continuous [2,34,37]. The mechanism and patterns of blood flow in the spectacle seem to guarantee visual acuity when snakes are active or presented with a visual stimulus and promote physiological shedding by avoiding desiccation of the spectacle layers during ecdysis [35,38]. Similar vascularization patterns of the spectacle or ocular scale have also been described in geckos, xantusiid lizards, and amphisbaenids [37,39].

**Figure 6:**
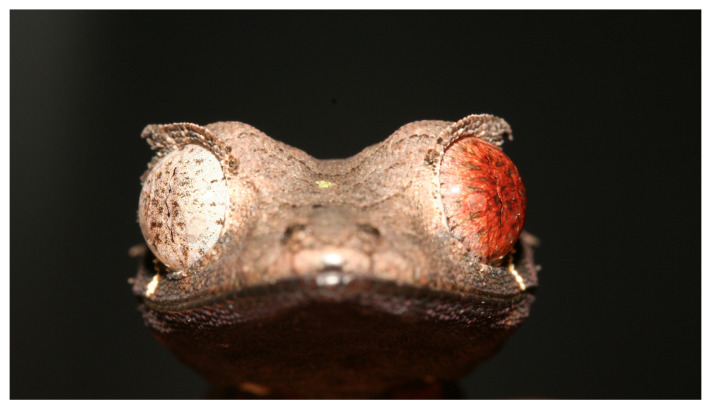
Unilateral dilation of iris vessels in the left eye of a satanic leaf-tailed gecko (*Uroplatus phantasticus*) can occur as a transient physiological phenomenon.

**Figure 7:**
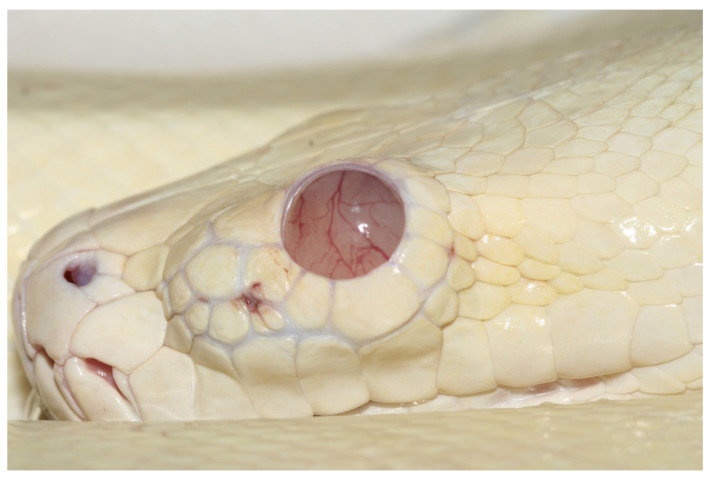
Engorged spectacular vessels in a *Burmese python* (*Python bivittatus*) with chronic pseudopubhthalmos. Besides overfilling of the subspectacular space, prominent swelling of the periocular and facial region can be noted.

Clinically, it is important to differentiate between a normal vascularization pattern, also during different stages of the shedding cycle, and neovascularization of the cornea, as well as physiological dilatation of iris vessels (Figure 6) or reactive dilation in case of uveitis. Engorged spectacular vessels might be seen in the chronic stages of pseudopubhthalmos or subspectacular abscesses (Figure 7) [31].

## 3. The Lacrimal System in Squamates

In snakes, lacrimal glands are absent; however, non-Harderian glandular structures that empty into the subspectacular space have been identified in boid species and parts of the epithelium of the subspectacular space might be secretory [6,40]. The serous Harderian gland of snakes is located in the dorsonasal orbit, medial and posterior to the eye and contains several ductules that drain into a lacrimal sac or ampulla that communicates with the ventral portion of the subspectacular space [5,41,42]. In general, saurian species with spectacles also lack lacrimal glands (except for members of the *Ablepharus* genus; Table 1) and their Harderian gland is located in the nasal aspect of the orbit. In geckos, it has been demonstrated that it is a seromucous gland that secretes proteins and lipids [3,26]. The Harderian gland is primarily responsible for the production of the tear film but might also play a role in vomeronasal olfaction [26,43].

In snakes, a single punctum is located in the ventromedial medial fornix of the subspectacular space and continues in the lacrimal duct [5,6,40,41]. After the duct passes through the lacrimal foramen, lateral to the prefrontal bone it shows a circuitous course rostrally and medially between the caudal aspect of the maxillary and palatine bones. Next, it runs between the vomer and the hypochoanal cartilage and exits in the oral cavity, medial to the opening of the duct of the vomeronasal organ [6,41]. Based on a computed tomographic anatomical study of the ophidian lacrimal system, the complexity of the tortuous course of the lacrimal duct varies between snake species [41]. This variation may be pertinent to predispositions of certain snake taxa towards the development of disorders of the subspectacular space, in particular pseudobuphthalmos and subspectacular infections, and also has implications towards the treatment and prevention of these disorders [41].

## 4. Ecdysis and Healing of the Spectacle

**Figure 8:**
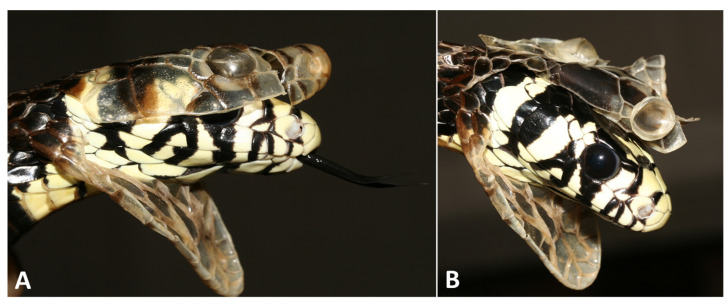
Ecdysis in a chicken snake (*Spilotes pullatus*). (**A**) Sloughing of the skin in a healthy snake should always occur in a single piece and include the spectacles. (**B**) The shed spectacle and its transition to the sloughed skin of the head are clearly visible and resemble a cast of the newly formed spectacle.

**Figure 9:**
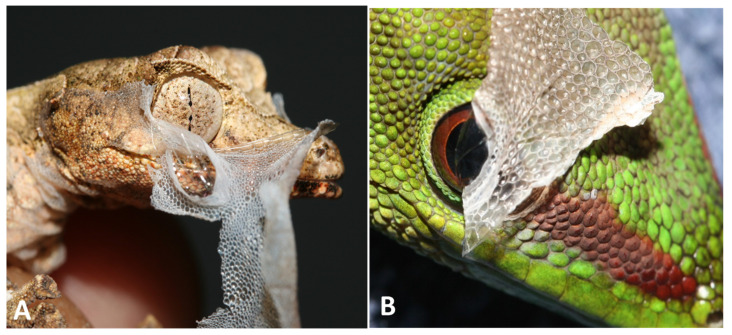
In most spectacled lizards, the renewal of the skin is a more gradual process in comparison with snakes, but the actual sloughing of the skin occurs in a short period of time. The spectacle should always be released together with the shed of the head as seen in (**A**) a satanic leaf-tailed gecko (*Uroplatus phantasticus*) and (**B**) a Madagascar day gecko (*Phelsuma madagascariencis*).

Ecdysis is the periodical process of renewal of the outer layer of the epidermis of the entire skin, including the spectacle [44,45,46]. In most squamates, this is a discontinuous, cyclic process. During the cleavage phase, the old skin separates from the newly formed layer by enzymatic activity of the lymphatic fluid. In snakes, the skin often appears dull or has a blueish hue during the renewal phase. This is most obvious in the spectacle as the result of accumulation of old and new layers of the epidermis causing opacification [44,45,46,47,48,49]. Curiously, this phenomenon does not occur or is almost unremarkable in lizards that have spectacles [2]. Shortly before the actual shed, the spectacle and skin of snakes regain their normal appearance as the lacunae of the stratum intermedium are broken down [47]. If a normal shedding cycle occurs, snakes shed their entire skin in a single piece (Figure 8 and Figure 9). Most lizards that have spectacles show a more gradual renewal process of the skin in comparison to snakes but will eventually also slough the old skin in a short period of time [48,49].

**Figure 10:**
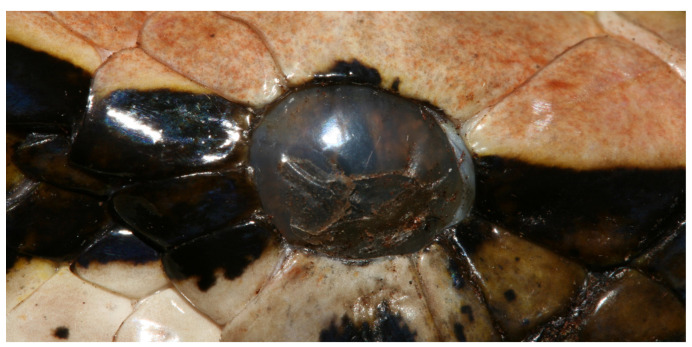
Appearance of the spectacle in a Burmese python (*Python bivittatus*) following a first shed after performing a partial spectaculectomy.

The clinical and histological healing of the spectacle was studied in ball pythons (*Python regius*) [50]. Twenty-four hours after experimental removal of a quadrant of the spectacle, infiltration of inflammatory cells and the development of a proteinaceous plug are noted at the cut edges of the spectacle. After 7 to 10 days, a proteinaceous crust bridges the spectacle defect. As the cellular inflammatory component gradually decreases, the deeper germinal epithelium becomes hyperplastic and covers the defect, whereas a prominent vascular response is present. After 21 days, the regenerated spectacle shows a normal vascular pattern beneath the crust. Initially, the thickness and transparency of healed spectacles may show considerable variation, and a normal appearance with a restored subspectacular space is mostly regained after one or more shedding cycles (Figure 10) [50]. The development of the proteinaceous crust is considered as a highly important step in the healing process as it acts as a scaffold for the regenerating epithelium of the spectacle [50].

Regeneration of the spectacle has been described in two snake species for the treatment of a near-complete spectaculectomy or spectacle avulsion by use of porcine small intestinal submucosal or amniotic membrane grafting combined with application of topical ointments [51,52]. In both cases, full regeneration of the spectacle following two or more shedding cycles was observed; based on these results, grafts are considered to serve as a scaffold for the healing of larger or complete spectacular defects [51,52].

## 5. Clinical Examination of the Spectacle and Subspectacular Space

Assessment of the spectacle and subspectacular space should always be included as a part of the general clinical examination of spectacled squamates [46,48]. Evidently, the presence of the spectacle excludes the application of fluorescein dye stain of the cornea, measuring lacrimal secretion or intraocular pressure [31].

Although many abnormalities can be detected with the naked eye, the detection and in-depth assessment of moderate to discrete changes often require the use of a magnifying glass. Slit-lamp biomicroscopy is highly useful to obtain a magnified three-dimensional view of the spectacle and the subspectacular space, as well as the anterior segment of the eye and the lens if a normal opacity of the spectacle and lacrimal fluid in the subspectacular space is present [31]. Although the normal microvasculature of the spectacle can vary greatly, it can be assessed using a slit lamp [31,35,38,53].

**Figure 11:**
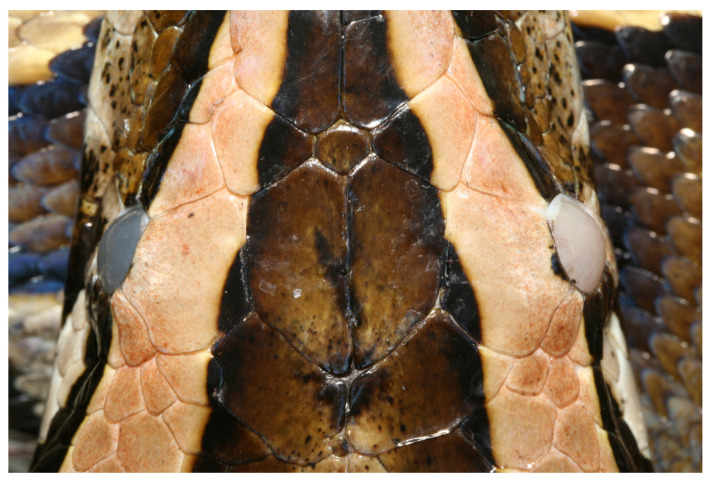
Bilateral opacification of the spectacle in a Burmese python (*Python bivittatus*). The blueish hue of the left spectacle is related to the renewal phase of the skin, whereas opacification of the right spectacle is caused by the presence of flocculent exudate in the ipsilateral subspectacular space due to bacterial infection.

Comparison of the left and right spectacle often allows the detection of mild distention of the subspectacular space or discrete changes in the opacification and the transparency of the lacrimal fluid (Figure 11). Any abnormality detected at the level of the spectacle or spectacular space should warrant appropriate testing and sampling. Systemic disease should be considered in case of bilateral lesions.

Optical coherence tomography (OCT) and scanning laser ophthalmoscopy have been used to visualize the spectacle, assess the thickness of the spectacle, and evaluate the subspectacular space [33,53]. In particular, OCT proves to be a valuable tool to assess the anterior segment structures of the spectacle [53,54].

During the clinical examination, and especially during the sampling and treatment of lesions of the spectacle and subspectacular space, appropriate local and/or general analgesic and anesthetic protocols should be used that are tailored on an individual basis and take into account the involved reptile species, anatomical structure, and lesion.

## 6. Retention of the Spectacle

**Figure 12:**
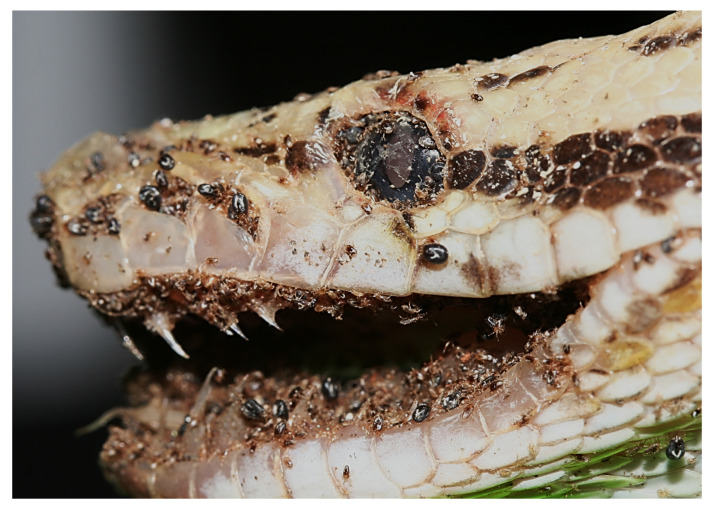
Extreme infestation with snake mites (*Ophionyssus natricis*) in a deceased ball python (*Python regius*).

Especially in snakes, retention of the spectacle is a commonly observed disorder. As the transition zone of the spectacle to the periocular zone is one of the body sites with the highest mechanical resistance, retention may also occur in healthy reptiles. If retained spectacles are part of generalized dysecdysis, this might be attributed to non-infectious causes, such as inappropriate environmental conditions (mainly inappropriate humidity levels or a lack of objects in the captive environment to rub against), as well as infectious causes, including ectoparasitosis (Figure 12) and bacterial, mycotic, or viral dermatitis. Malnutrition or generalized disorders, especially those resulting in dehydration and/or hypoproteinemia, may also result in impaired shedding, including spectacular retention [44,45,46,48]. The diagnosis and treatment of the primary causes of retained spectacles are indispensable parts of the treatment of this disorder.

**Figure 13:**
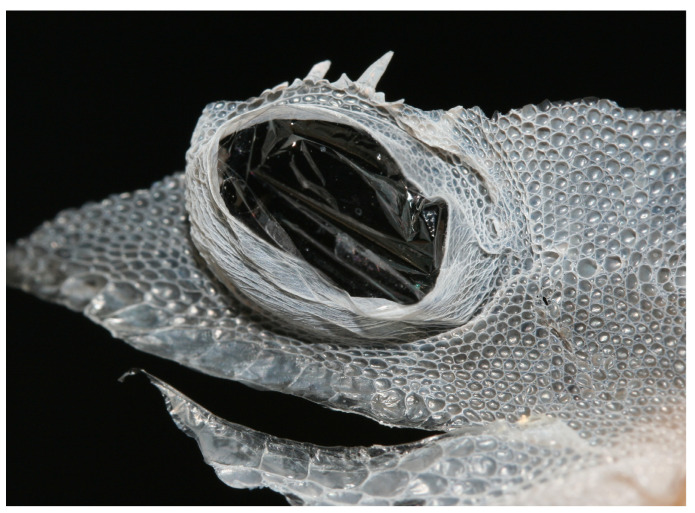
Examination of the shed skin from a northern spiny-tailed gecko (*Strophurus ciliaris*) confirms complete shedding of the spectacle.

Retention of the spectacle is easily diagnosed if several layers of shed spectacle have accumulated in contrast to retention of a single layer, especially shortly after sloughing, as this results in minimal changes in opacification. Whenever possible, the shed skin should be examined for the presence of the spectacles (Figure 13).

**Figure 14:**
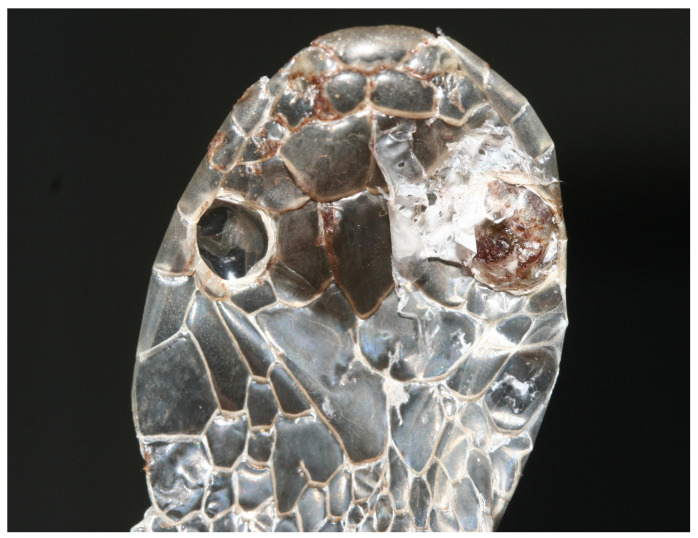
Fresh shed skin from the head of a corn snake (*Pantherophis guttatus*) detached a retained spectacle at the right side.

In some cases, the retained spectacle may be released spontaneously with the next shed (Figure 14).

**Figure 15:**
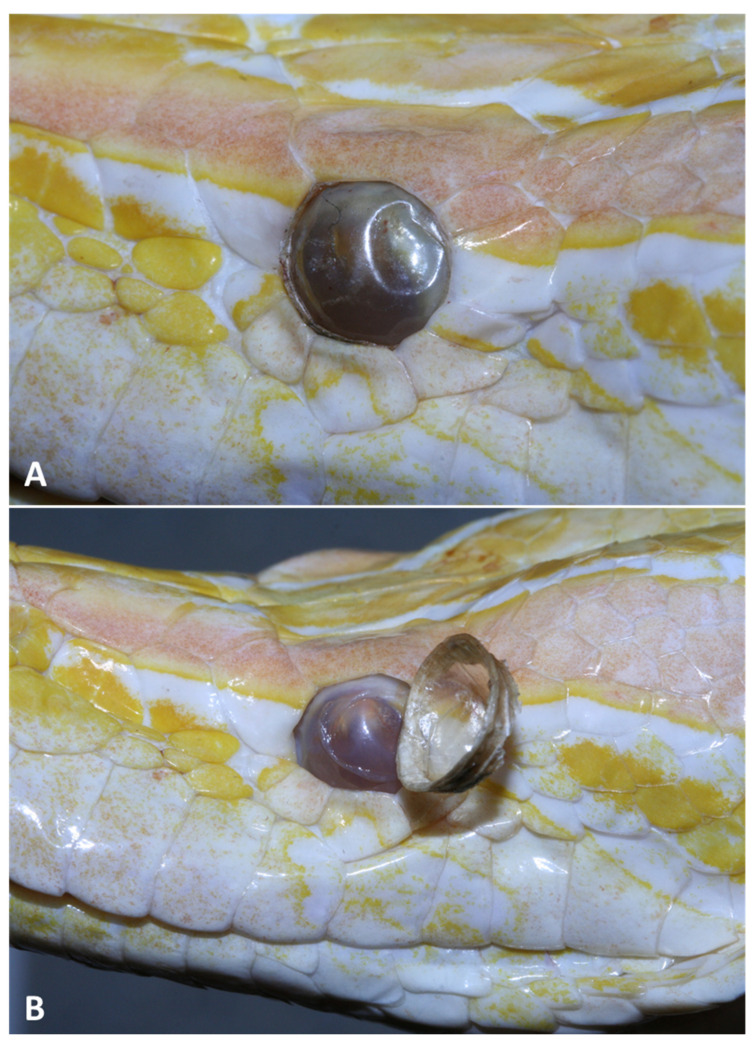
(**A**) Multiple retained layers of the spectacle in a Burmese python (*Python bivittatus*). (**B**) Release of the retained layers after a few days of topical treatment with ophthalmological ointments.

Multiple layers of retained shed often become very rigid and highly difficult to remove. Premature removal or aggressively peeling back the retained spectacle may damage the normal epidermal layer and in severe cases the subspectacular space and/or cornea might be exposed with the development of corneal ulceration, exposure keratitis, or even panophthalmitis [44,45,46,48]. Repeatedly putting the affected snake or lizard in lukewarm water baths for 10 to 15 min in combination with optimization of the environment and/or applying topical eye droplets, tear preparations, or ointments multiple times a day mostly allows the safe removal of retained spectacles (Figure 15) [44,45,46].

Hydrodissection is a technique that consists of irrigating saline between the spectacle layers using a 23-to-27-gauge catheter that seems to be quite effective to rapidly delineate and remove retained layers without the risk of removing the basal layer of the spectacle [31].

## 7. Indentation and Trauma of the Spectacle

Bilateral or unilateral indentations of the spectacle can be regularly seen in various snake species, notably ball pythons. In most cases these indentations are physiological, although they may also be associated with mild trauma, generalized skin disorders, or systemic diseases. Indentations of the spectacle will resolve quickly with the next shed unless underlying disease is present [44,45,46,47,48].

**Figure 16:**
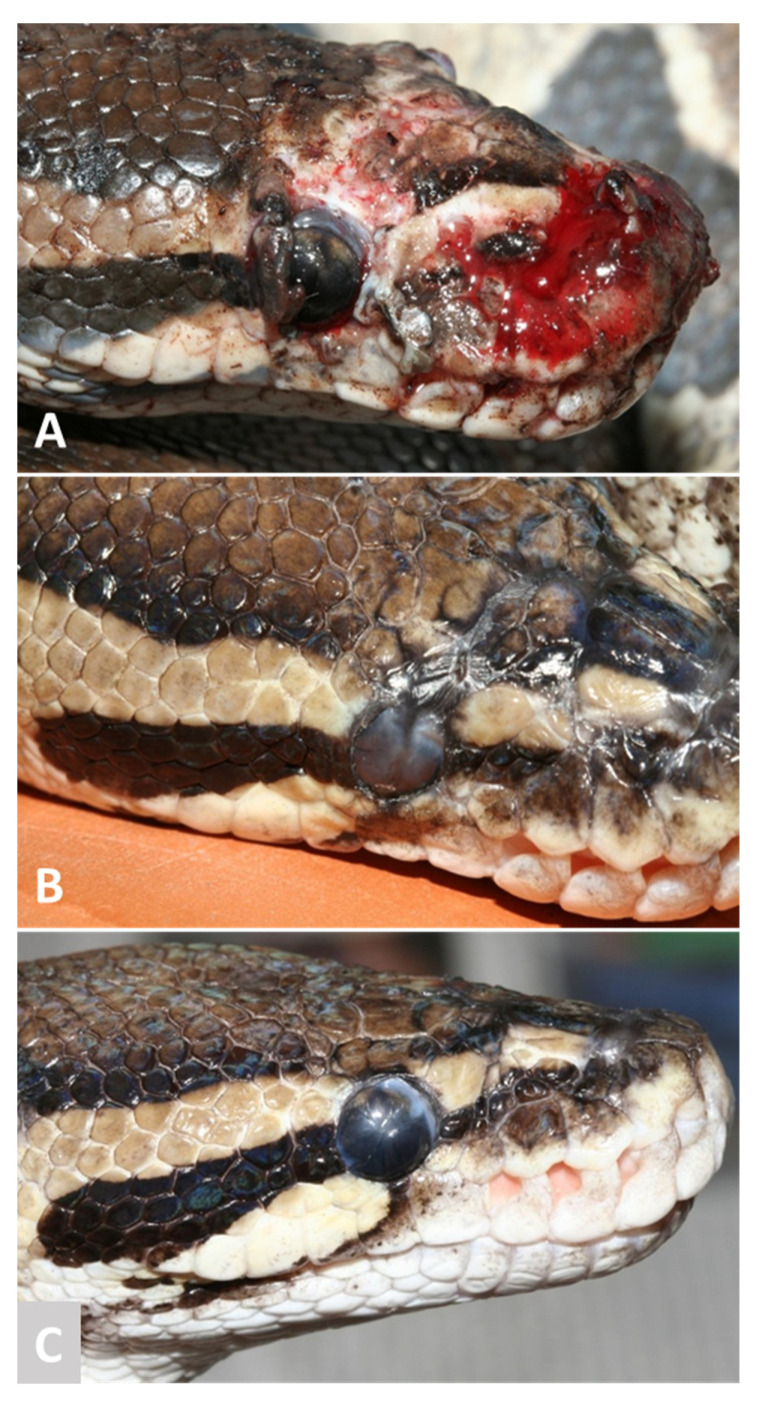
(**A**) Rat bite induced rostral trauma involving the spectacle in a ball python (*Python regius*). (**B**) Healing of the skin and spectacle following topical wound treatment and shedding (week 4). (**C**) Mild residual scarification of the skin and spectacle after a second shedding phase (week 16).

Injuries of the spectacle are most frequently seen as a consequence of iatrogenic trauma (e.g., inappropriate removal of retained spectacle layers), trauma from the environment, especially in snakes or lizards that show repetitive rubbing or explosive flight behaviour, or biting lesions caused by prey items (Figure 16) [48]. Often, this coincides with more extensive rostral trauma, dermal granulomas, and/or stomatitis. Although mild trauma and abrasions of the spectacle will often heal quickly with the next shedding phase, secondary bacterial or fungal infection might occur if the deeper layers of the spectacle are exposed [44,45,46,47,48].

It has been postulated that in cases of traumatic near-complete spectaculectomy or avulsion of the spectacle, spontaneous regeneration will not occur and intensive treatment, including the use of grafting, is necessary [31,51,52]. Based on our experiences, however, topical application of antimicrobial ointments until a first shed has occurred has a fair chance of resulting in complete regeneration of the spectacle and subspectacular space if the basal layers at the transition zone are still intact. Although the regenerated spectacle may initially appear wrinkled, it mostly regains its normal appearance after one or more additional shedding cycles.

## 8. Spectaculitis

**Figure 17:**
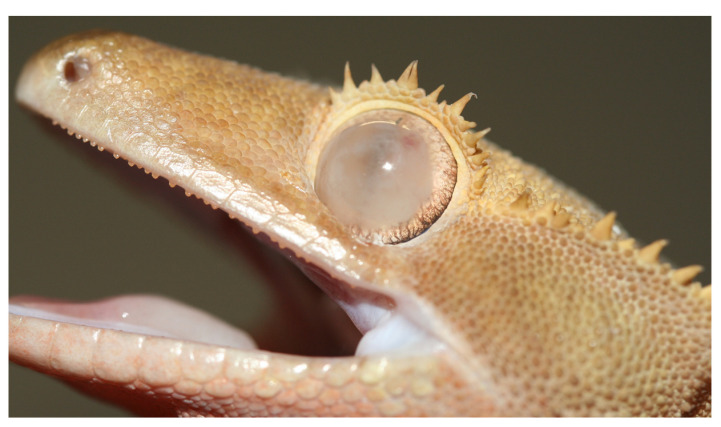
Spectaculitis associated with bacterial infection in a crested gecko (*Correlophus ciliatus*).

**Figure 18:**
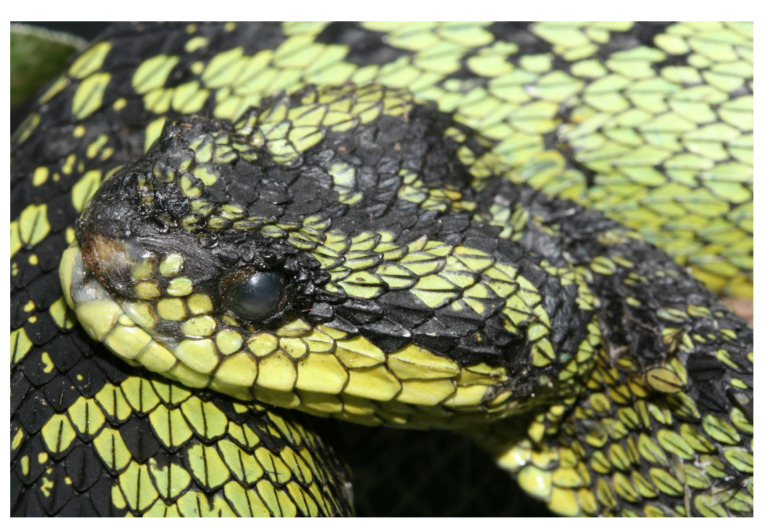
Dermatitis of the preocular and nasal skin extending to the medial part of the spectacle associated with opportunistic mycosis in a great lakes bush viper (*Atheris nitschei*).

Spectaculitis may be seen as a localized lesion (Figure 17) or be part of a generalized dermatoses, including those that are associated with systemic disease [29,31,55]. If spectaculitis coincides with dermatitis, a mutual etiology can often be revealed (Figure 18) [44,45,46,47,48].

Gram-positive bacteria are more commonly encountered in snakes with spectaculitis than in other diseases [28,56]. *Ophidiomyces ophiodiicola* has been reported as a cause of spectaculitis in both wild and captive snakes [57,58]. Rather anecdotally, the presence of spectacular urate depositions as well as cases of disseminated lymphoma and myeloproliferative disease with involvement of the spectacle have been reported in snakes [31]. The latter disorders may be associated with localized or generalized inflammation and opacification of the spectacle. Local or diffuse opacification of the spectacle may develop following exposure to excessively short UV-B wavelengths [31,59].

Histological features of spectaculitis include serocellular crust formation, epithelial and stromal edema, stromal neovascularization, and infiltration of inflammatory cells. In most cases, the stroma and outer epithelial layers are involved, whereas the inner epithelium only seems to be involved in severe cases of deep bacterial or mycotic infections [28,29]. If the inner epithelial layer is involved, cellular hypertrophy and hyperplasia may occur and necrotic epithelial cells may enter the subspectacular space [28,29].

**Figure 19:**
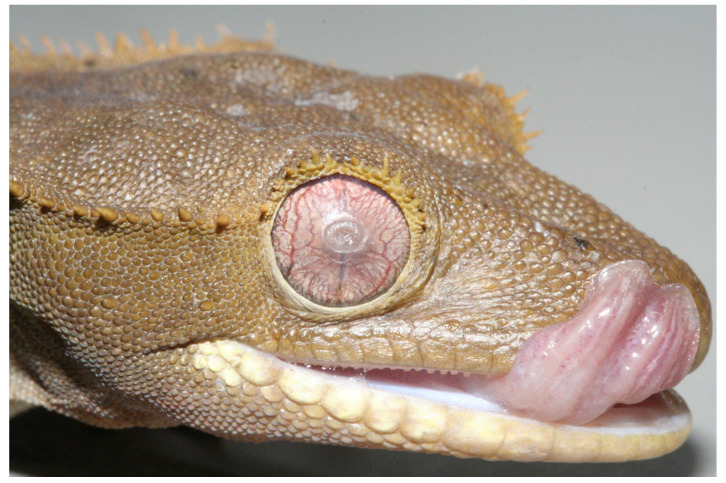
Ulcerative lesion located at the center of the spectacle in a crested gecko (*Correlophus ciliatus*).

*Correlophus* and *Rhacodactylus* species (Diplodactilydae) frequently present with localized, ulcerative lesions in the center of the spectacle (Figure 19). Although the exact etiology is unknown, the development of these lesions might be associated with opportunistic infection caused by bacteria that are part of the oral microbiota. Although most geckos clean and moisturize the spectacle by use of the tongue, *Correlophus* and *Rhacodactylus* species seem to be predisposed to such infections if the integrity of the epidermal barrier of the spectacle is impaired due to microtrauma, lacerations, inappropriate environmental conditions, and/or secondary changes due to nutritional deficiencies. Especially during the vitellogenic phase of the reproductive cycle, female crested geckos seem to be particularly prone to the development of these typical lesions of the spectacle. In most cases, lesions resolve following application of topical vitamin A and/or antimicrobial ointments followed by renewal of the spectacle, although fibrosis might develop and recurrent episodes are commonly observed.

## 9. Pseudobuphthalmos

**Figure 20:**
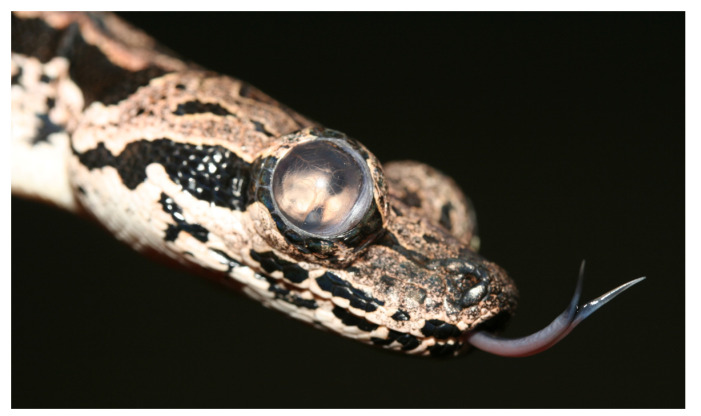
Pseupdobuphthalmos in a neonate Dumeril’s boa (*Acrantophis dumerili*) that is presumably related to developmental abnormalities in the lacrimal drainage system.

**Figure 21:**
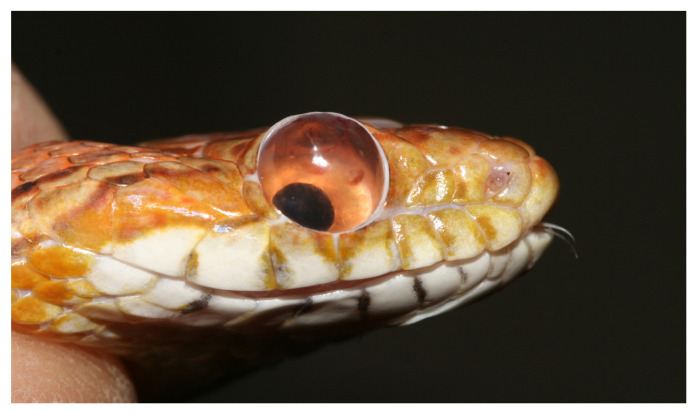
Pseudobuphthalmos presenting as a severe distention of the subspectacular space related to obstruction of the lacrimal duct in a corn snake (*Pantherophis guttatus*).

**Figure 22:**
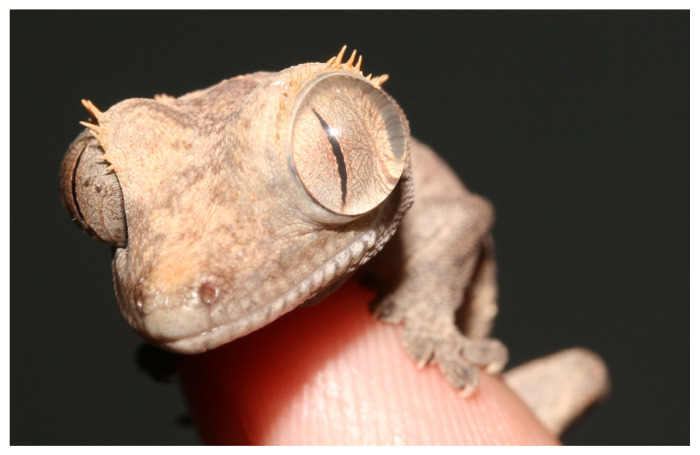
Pseupdobuphthalmos in a juvenile crested gecko (*Correlophus ciliatus*) resulting from developmental abnormalities of the lacrimal drainage system.

Pseudobuphthalmos, also referred to as bullous spectaculopathy, results from obstruction of the lacrimal drainage system and is seen in snakes (Figure 20 and Figure 21) as well as lizards (Figure 22) [11,31,47].

**Figure 23:**
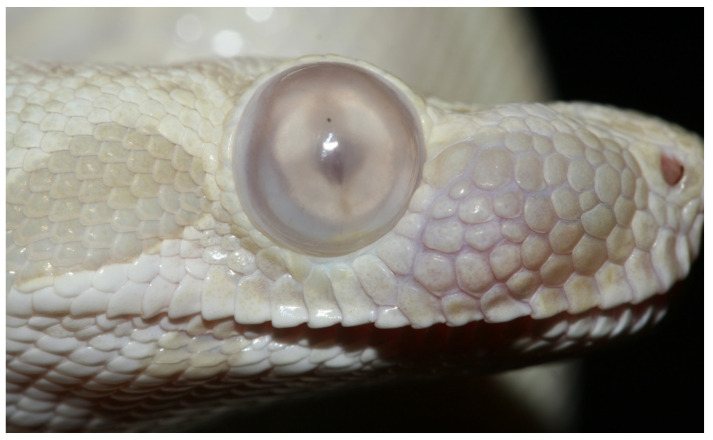
Pseudobupthalmos in a juvenile boa constrictor (*Boa constrictor*) combined with swelling of the facial region associated with ascending *Pseudomonas aeruginosa* infection via the lacrimal duct. If left untreated, subspectacular infection and abscessation might develop.

In comparison to subspectacular infection, pseudobupthalmos is more frequently seen in young animals and it may occur uni- or bilaterally. Typically, the excessive lacrimal fluid in the subspectacular space is clear and the spectacle as well as the ocular globe show a normal appearance. In severe cases, swelling of the facial and medial periocular region (Figure 23) may occur [11,47,60].

**Figure 24:**
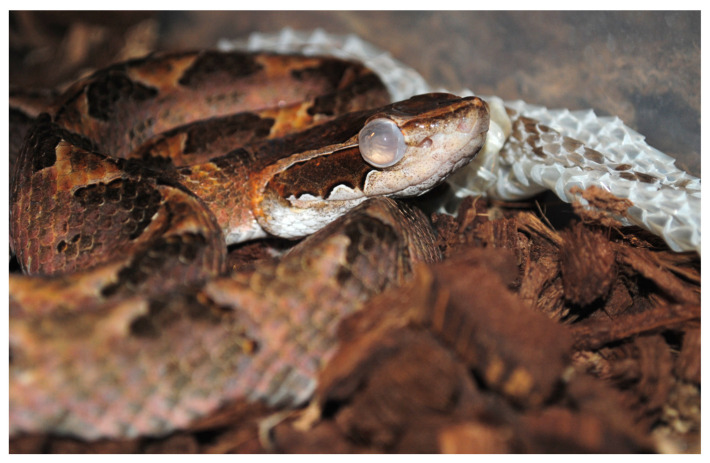
Idiopathic congenital pseudobupthalmos in a neonate Malayan pit viper (*Calloselasma rhodostoma*) shortly after the first shed after hatching. A morbidity of approximately 50% was witnessed within the clutch.

Congenital pseudobupthalmos in neonates is possibly associated with developmental abnormalities of the lacrimal duct [60]. In ovoviviparous snakes, the authors have witnessed cases where up to 60% of the neonates from a single clutch were affected (Figure 24). In some cases, development into subspectacular abscesses was observed.

**Figure 25:**
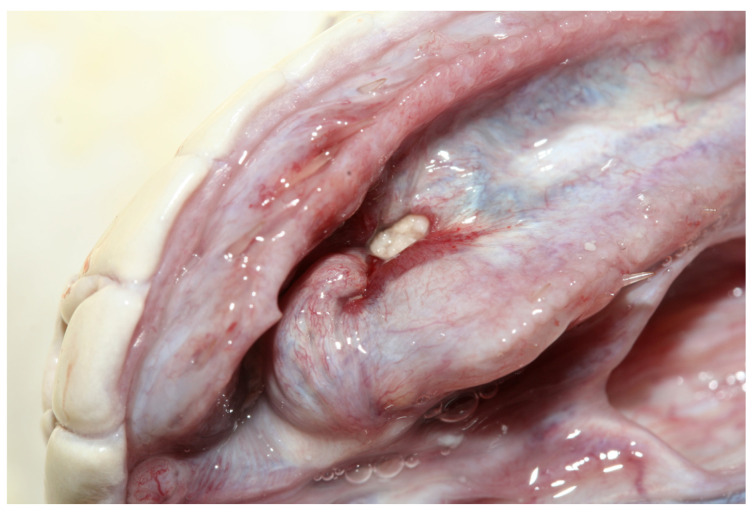
Debris obstructing the combined orifice of the lacrimal duct and vomeronasal organ in the roof of the mouth in a Burmese python (*Python bivittatus*) with pseudo-buphthalmos.

**Figure 26:**
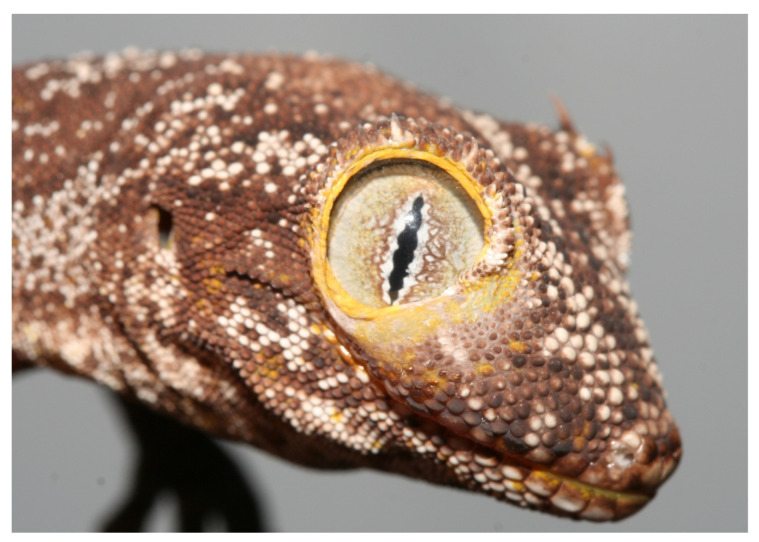
Medial peri-ocular and facial swelling associated with ascending bacterial infection of the lacrimal duct in a northern spiny-tailed gecko (*Strophurus ciliaris*).

Mild pseudobupthalmos can be seen shortly before the actual sloughing of the shed in snakes. This might be attributed to obstruction of the drainage at the lacrimal punctum caused by the accumulation of spectacle layers in the ventromedial subspectacular space [31]. Although those cases mostly resolve spontaneously, recurrence might be observed with each shedding cycle [61]. Other causes of pseudobuphthalmos also include direct or indirect blockage along the course of the lacrimal drainage such as caseous stomatitis with debris obstructing the drainage opening (Figure 25) or ascending infection of the lacrimal duct (Figure 26), abscesses, granulomas, foreign objects (e.g., substrate), trauma, or neoplasia located in the roof of the mouth or the facial tissues [11,31,47]. Especially in lizards, hypovitaminosis A with the development of metaplasia of the lacrimal duct and subsequent (sub)obstruction of the duct should be considered [48]. Similar predispositions and etiologies are commonly associated with dacryocystitis in, e.g., rabbits and cats in which the lacrimal duct also shows pronounced tortuosity [31,41].

In case of pseudobuphthalmos, sampling of the clear fluid from the subspectacular space mostly yields negative results following microbiological examination [31]. Pseudobuphthalmos, however, can be an early presentation of ascending infection of the lacrimal duct from the oral cavity and might eventually lead to the development of subspectacular abscessation once infection has reached the subspectacular space. Secondary infection may occur if the distended spectacle is traumatized or perforated, mostly in cases where affected snakes or lizards show reactive and repetitive rubbing behavior. Detection and elimination of the primary etiology is fundamental towards the successful treatment of pseudobophtalmos. Congenital pseudobuphthalmos that is related to developmental disorders has a poor prognosis and, theoretically, conjunctivolateralostomy is often the single treatment option [11,31,47,60].

**Figure 27:**
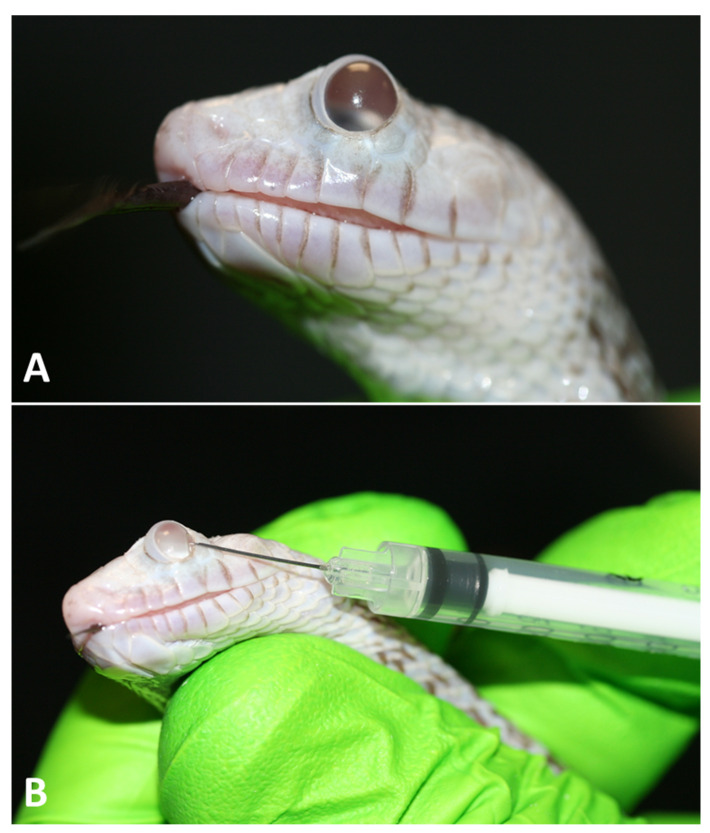
(**A**) Pseudobuphthalmos in a gopher snake (*Pituophis catenifer*). (**B**) Drainage of the subspectacular space via punction through the spectacle.

As the fluid in the subspectacular space is often pure lacrimal fluid, percutaneous aspiration of the fluid by inserting a needle between the periocular scales or aspiration following puncture through the spectacle (Figure 27) is highly feasible. Repeated aspiration of the fluid and flushing of the subspectacular space may be necessary. Unusually, a partial spectaculectomy needs to be performed as described for treating subspectacular abscesses. If the spectacle is persistently and severely distended, it may be considerably stretched and become wrinkled and collapse once the excessive volume of subspectacular fluid is alleviated [31].

**Figure 28:**
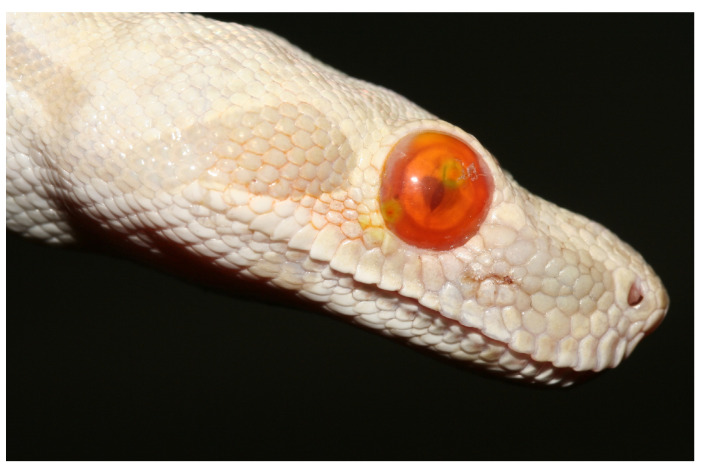
Boa constrictor (*Boa constrictor*) following percutaneous injection of a 1:5 balanced salt dilution of 10% fluorescein into the subspectacular space to ascertain patency of the lacrimal drainage system following treatment for pseudobuphthalmos.

Successful treatment of pseudobuphthalmos can be confirmed by assuring restored patency of the lacrimal duct. The latter may be performed by injection of a 1:5 balanced salt dilution of 10% fluorescein into the subspectacular space, either percutaneously (Figure 28) or through the spectacle [41]. If these routes seem ineffective, it might be useful to attempt retrograde flushing via the drainage orifice of the lacrimal duct in the roof of the mouth, although this might induce iatrogenic infection from the oral cavity.

## 10. Subspectacular Infection

**Figure 29:**
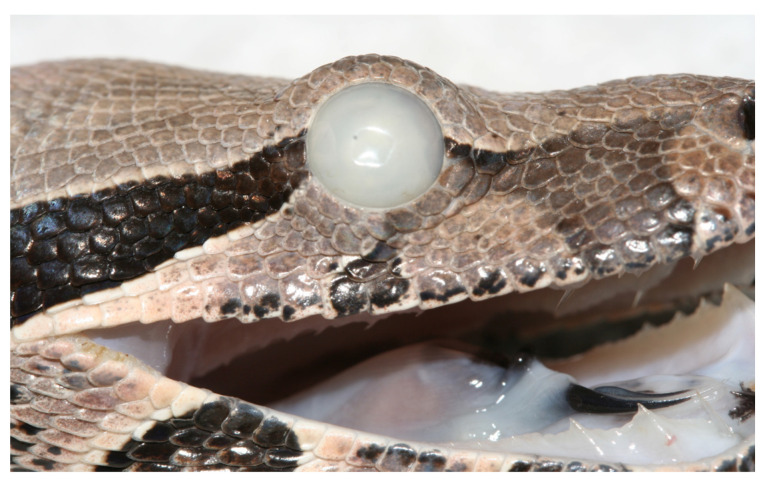
Subspectacular infection associated with bacterial infection in a boa constrictor (*Boa constrictor*). The subspectacular space is filled with flocculent exudate.

**Figure 30:**
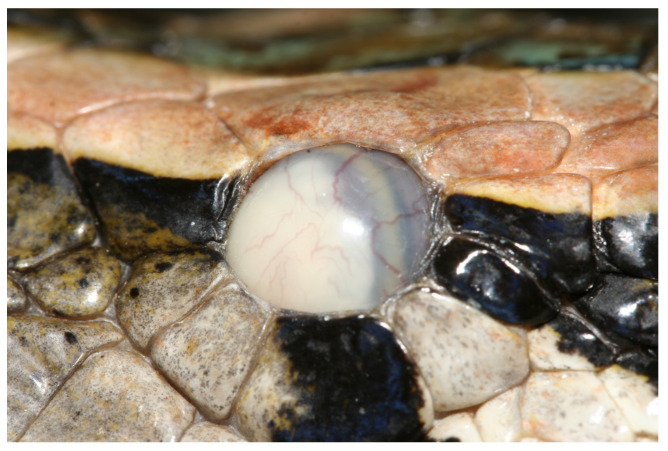
Flocculent exudate causing partial opacification of the subspectacular space and mild engorgement of the blood vessels of the spectacle in a Burmese python (*Python bivittatus*).

**Figure 31:**
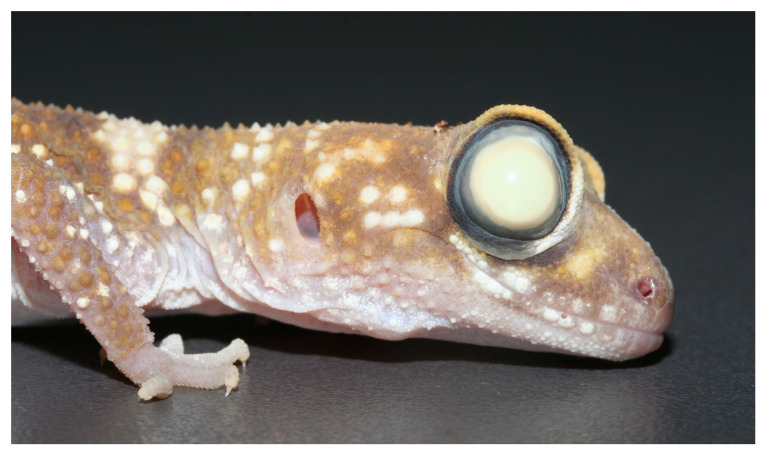
Thick-tailed gecko (*Underwoodisaurus milii*) with subspectacular abscess associated with bacterial infection.

Subspectacular infection is presumably the most common disorder of the spectacle and its drainage system in reptiles. Boa constrictors (*Boa constrictor*) (Figure 29) and Burmese pythons (*Python bivittatus*) (Figure 30) seem to be especially predisposed to the development of subspectacular infection, although the disorder is also diagnosed in colubrids and to a lesser extent in viperids and lizards (Figure 31). Although subspectacular infection might resemble pseudobuphthalmos in the earliest stages, the fluid in the subspectacular space mostly becomes nontransparent with the appearance of flocculent exudate as seen in hypopyon of the anterior eye chamber [11,31,44,47,61,62].

**Figure 32:**
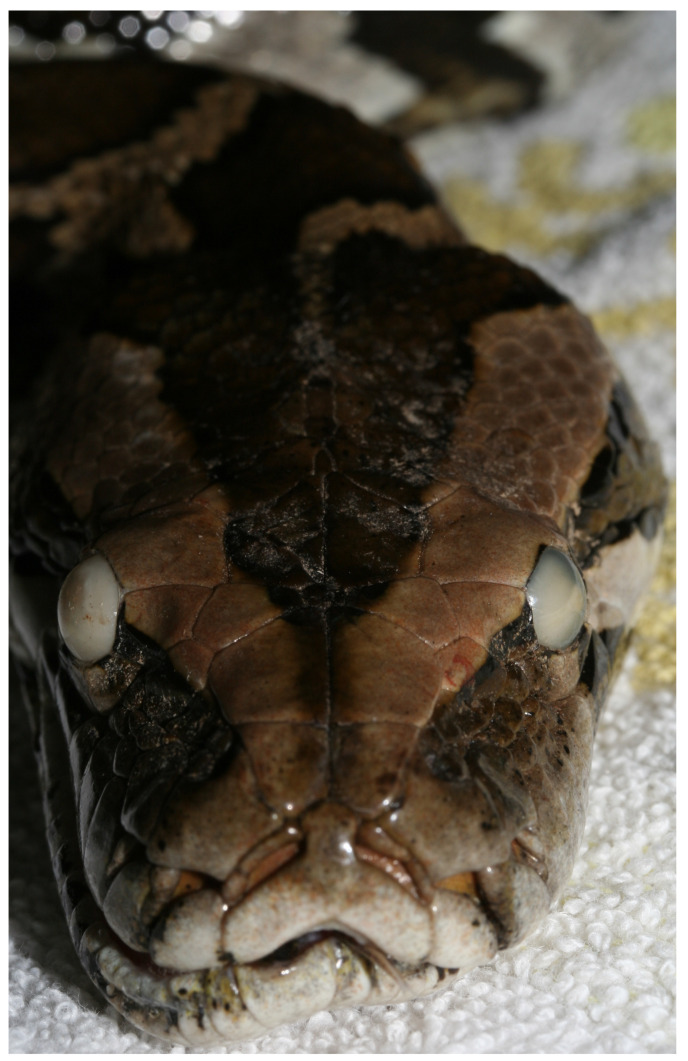
Bilateral subspectacular abscesses as seen in this Burmese python (*Python bivittatus*) is a relatively unusual presentation and should warrant the inclusion of possible systemic bacterial infection as a primary etiology.

**Figure 33:**
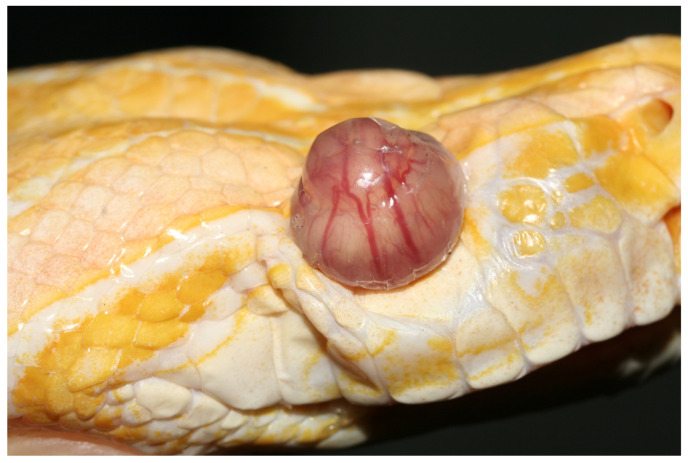
Prominent engorgement of blood vessels as well as severe distention and deformation of the spectacle as observed in a Burmese python (*Python bivittatus*), indicating chronic subspectacular abscessation.

**Figure 34:**
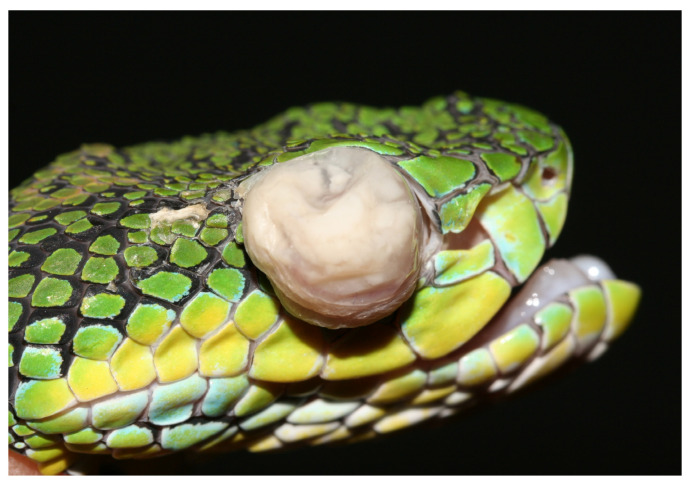
Extremely chronic subspectacular abscess in a Schultze’s pit viper (*Trimeresurus schultzei*). Avulsion of the spectacle may occur if manipulated because of the chronic inflammatory changes.

If bilateral infection is present (Figure 32), systemic infection should be considered as a primary etiology. The amount of exudate, thickening, and opacification of the spectacle as well as engorgement of the blood vessels (Figure 33) are correlated with the severity and/or chronicity of the disorder. In chronic stages or cases of severe distension of the spectacle, the spectacle may show a whitish appearance and become extremely fragile because of impaired vascularization (Figure 34). In those cases, the spectacle might collapse and wrinkle or might even detach at the level of the transition zone when manipulated. When left untreated, subspectacular infection might extend to the periocular and facial region [11,31,44,47,61,62].

**Figure 35:**
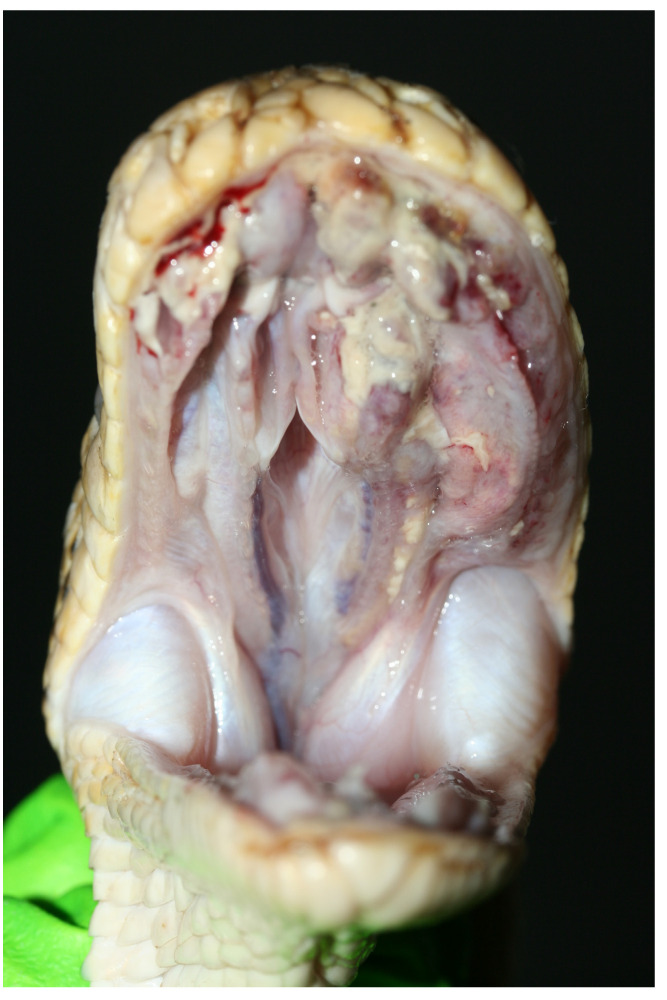
Caseous stomatitis as seen in this reticulated python (*Malayopython reticulatus*) is commonly seen in snakes with subspectacular infection as associated bacteria as well as flagellated protozoa may reach the subspectacular space following ascending infection via the lacrimal duct.

Except for rare cases where infection enters the subspectacular space through perforation of the spectacle or results from systemic infection, ascending infection from the oral cavity via the lacrimal duct is the predominant cause of subspectacular infection [11,44,45]. It is of fundamental importance to diagnose and eliminate facilitating or primary causes of subspectacular infection in reptiles, such as upper respiratory tract and oropharyngeal infection, in particular caseous stomatitis (Figure 35) and esophagitis. Especially in boa constrictors, these infections are commonly seen as a comorbidity to inclusion body disease caused by divergent reptarenaviruses [31,63].

In almost all cases, bacterial isolates can be cultured from subspectacular abscesses and generally comprise Gram-negative bacteria, such as *Pseudomonas aeruginosa*, *Aeromonas* species, and *Salmonella* species, but infection caused by *Clostridium perfrigens* or methicillin-resistant *Staphylococcus* sp. infection has also been reported [11,61,62,64,65]. Interestingly, combined infection of the subspectacular space with flagellated protozoan parasites, presumably *Tritrichomonas* and *Monocercomonas* species, are frequently seen in snakes with bacterial subspectacular abscesses and flagellated protozoan infection has also been demonstrated in geckos with subspectacular abscesses [11,66]. Although the role in the etiopathogenesis of subspectacular abscesses remains unclear, it is certain that these protozoal parasites also reach the subspectacular space via ascending infection from the oral cavity, as the parasites can also be detected in the oropharyngeal cavity in most of these cases. Less frequently, *Serpentirhabdias*-species eggs and nematode worms have been detected in mostly bilateral subspectacular lesions in snakes [67,68].

**Figure 36:**
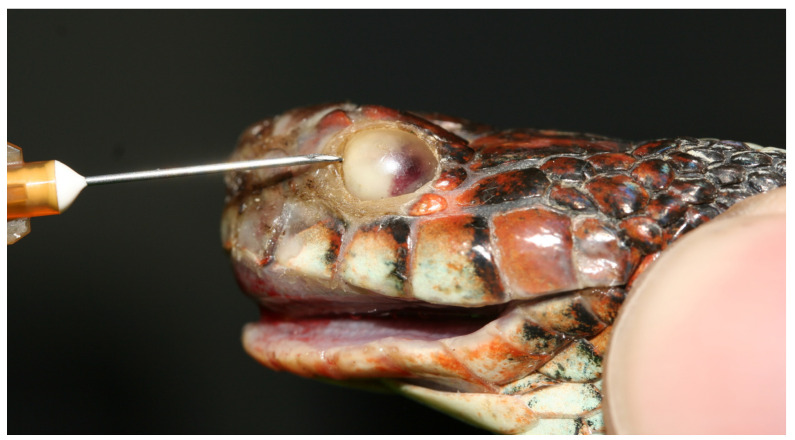
Sampling of a subspectacular abscess in a red-spotted garter snake (*Thamnophis sirtalis concinnus*) via fine needle aspiration through the spectacle.

**Figure 37:**
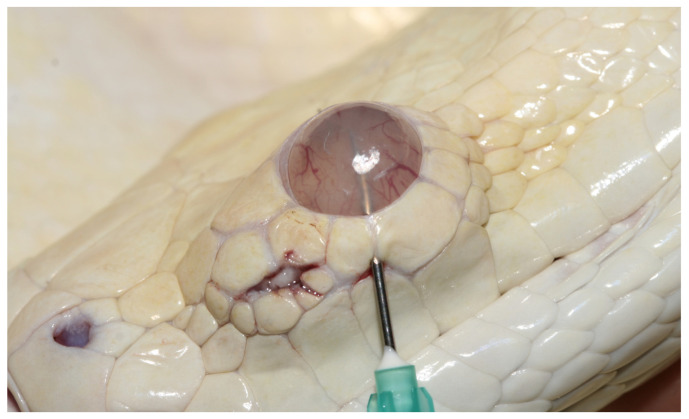
Sampling of a subspectacular abscess in a Burmese python (*Python bivittatus*) via fine needle aspiration by insertion of the needle in between the periocular scales.

Although subspectacular infection is easily diagnosed based on the clinical appearance, the management of this disorder should always include the search for possible primary systemic infection or causes of generalized or localized immunosuppression. The collection of exudate from the subspectacular space (Figure 36 and Figure 37) for cytology (wet preparations, Gram staining) and microbiological culture (including mycology), including susceptibility testing, is of fundamental importance. Molecular testing might be necessary to demonstrate fastidious organisms (e.g., *Chlamydia* spp. and *Chlamydia*-like organisms, *Mycobacterium* spp.). As the flocculent exudate mostly prevents the collection of qualitative samples by FNA (Figure 34), it is advisable to collect samples when creating a partial speculatectomy opening, as almost in all cases this is an integral part of the treatment [11,31,47,61,62,64].

**Figure 38:**
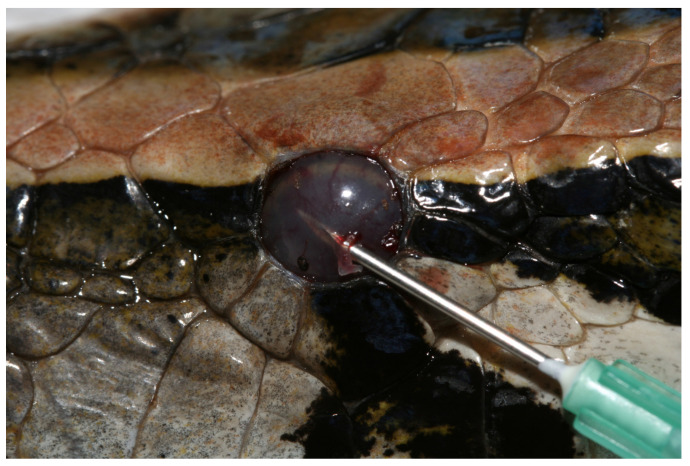
Insertion of a 21-gauge needle into a Burmese python (*Python bivittatus*) through the spectacle may allow aspiration of exudate from the subspectacular space and is also used as a first step in performing a partial spectaculectomy.

**Figure 39:**
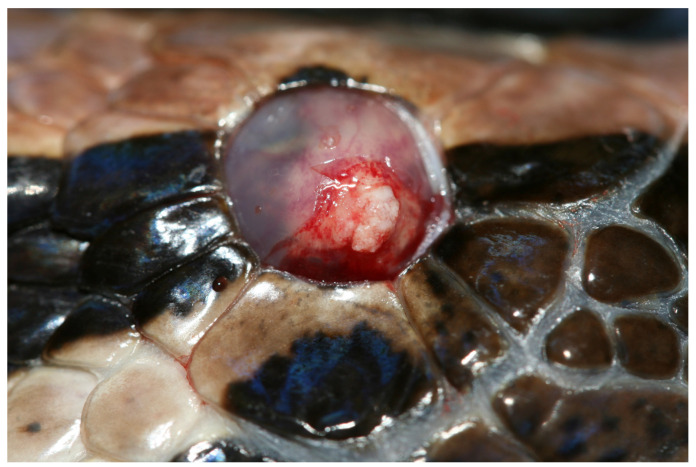
Partial spectaculectomy in a Burmese python (*Python bivittatus*) with a subspectacular abscess allows removal of the exudate from the subspectacular space.

Conventionally, performing partial spectaculectomy entails the creation of a small 25-to-30-degree wedge in the ventrolateral quadrant of the spectacle, as this minimizes the risk of damaging the drainage punctum [41]. In small-sized reptiles, it might be advisable to use an operation microscope to avoid iatrogenic damage to the cornea. Introduction of a needle into the subspectacular space is recommended, followed by the use of Wescott tenotomy scissors to excise the spectacle window (Figure 38 and Figure 39).

Once the wedge has been created, the debris can be removed if complete aspiration was not feasible and topical antimicrobial administration can be performed. Treatment should be based on antimicrobial susceptibility testing and irrigation of the subspectacular space for an average 5 to 7 days is recommended [11,31,63,64]. Although crust formation is considered as an integral part of the healing process of the spectacle [50], it will prevent topical applications from reaching the subspectacular space. Consequently, it is recommended to avoid total dissolution of the crust during repeated irrigation of the subspectacular space or to perform this percutaneously [31,50].

**Figure 40:**
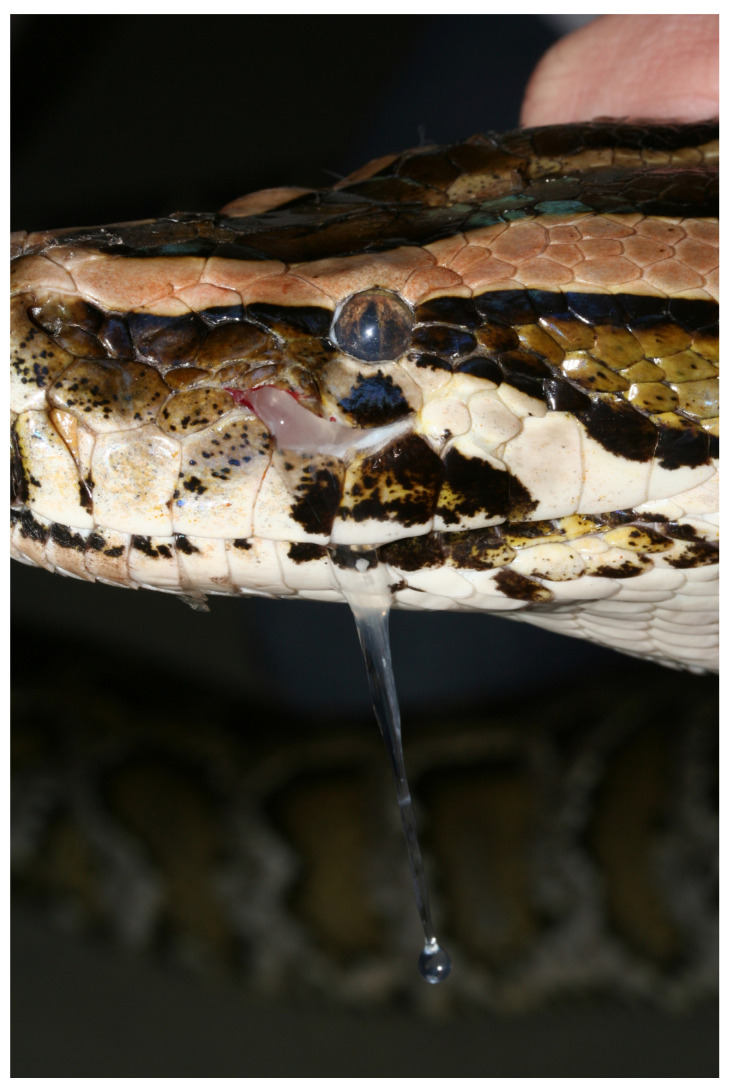
Whenever subspectacular infection of the spectacle has extended to the periocular and/or facial region, making an incision in between the scales is often required to allow drainage and local treatment of the subcutaneous infection.

As geckos moisten and clean their spectacles using their tongue, ingestion of excessive amounts of topical applications should be avoided. If keratomalacia is present, topical sodium EDTA may be useful to reduce collagenase activity [31]. If flagellate infection is confirmed, especially when concurrent oropharyngeal infection is present, per oral antiprotozoal treatment should be initiated [66]. In case exudative subspectacular infection has extended into the periocular and facial subcutaneous tissues, it might be necessary to surgically debride and flush these lesions (Figure 40), similar to what is performed during the treatment of subcutaneous abscesses or granulomas in reptiles.

**Figure 41:**
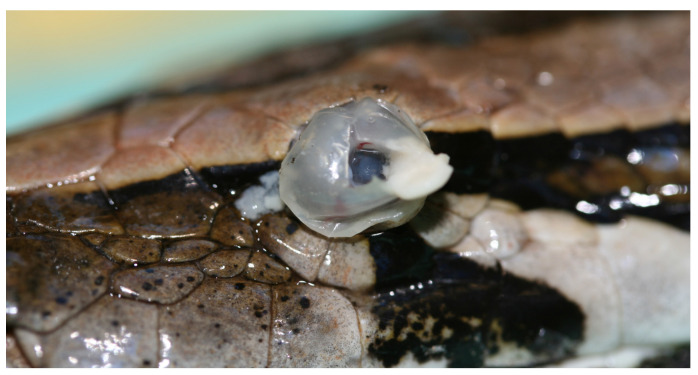
Collapse and wrinkling of the spectacle after performing a partial spectaculectomy and removal of the exudate in a Burmese python (*Python bivittatus*) are frequently seen in cases of chronic infection.

Once the exudate has been removed from the subspectacular space and infection has been eliminated, the distention of the spectacle often decreases quickly and the normal appearance of the subspectacular space and fluid restores gradually. As described for pseudobuphthalmos, the spectacle may collapse and wrinkle if chronic and severe distention occurred (Figure 41), and it is highly advisable to confirm patency of the lacrimal duct during or at the end of the treatment.

**Figure 42:**
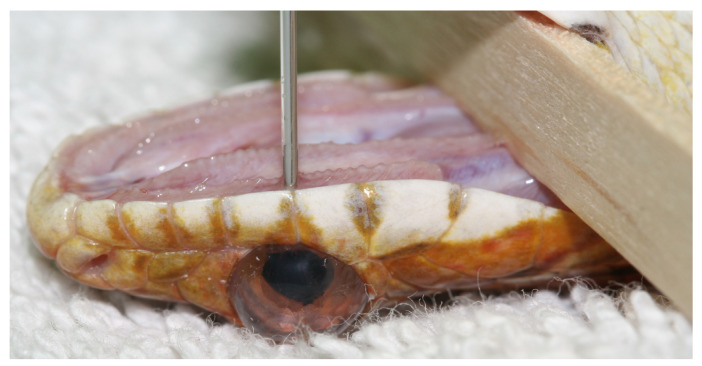
Conjunctivoralostomy performed in a corn snake (*Pantherophis guttatus*) with pseudobuphthalmos comprises the creation of a new channel. In this case, a metal cannula is advanced from the fornix between the lateral maxillary teeth and the facial skin to the ventral subspectacular space as the initial step of the procedure.

If persistent pseudobuphthalmos occurs, e.g., in cases of developmental disorders causing lacrimal duct obstruction or secondary to subspectacular infection, attempts can be made to recreate lacrimal drainage. The latter procedure is referred to as conjunctivoralostomy and comprises the creation of a new channel with a metal cannula or an appropriately sized needle from the fornix between the lateral maxillary teeth and edge of the oral cavity to the ventrolateral part of the spectacle [69]. The latter may be performed starting from the subspectacular space via a 30° wedge incision of the spectacle or from the fornix (Figure 42). Next, a 28-gauge silastic tube is threaded through the channel and fixed with sutures at its dorsal location and ventrally to the facial skin via a small incision in the labial scales. The tube has to be left in situ for at least 8 weeks in order to allow epithelialization around the tube. The latter is only feasible in reptiles with a considerable size, and postoperative fibrosis and occlusion of the newly created drainage duct are seen as the main complications [69].

## 11. Conclusions

The reptilian spectacle is considered as the most sophisticated type of spectacle and is one of the most distinguishable traits of snakes and certain lizard species. In general, reptiles with a spectacle lack lacrimal glands. The tear film that fills the subspectacular space is produced by the Harderian gland and drains to the oral cavity via the lacrimal duct. Retention of the spectacle, spectaculitis, and injuries of the spectacle are commonly observed disorders. Especially in snakes, the complexity of the tortuous course of the lacrimal duct may vary between species and may predispose certain species towards the development of disorders of the subspectacular space, in particular pseudobuphthalmos and subspectacular infection. These conditions are caused by blockage of the lacrimal drainage system and ascending infection of the subspectacular space from the oropharyngeal cavity, respectively. A sound knowledge of the anatomy and physiology of the spectacle and its drainage system allows the establishment of an adequate diagnostic and therapeutic approach for these disorders.

## Figures and Tables

**Figure 2:**
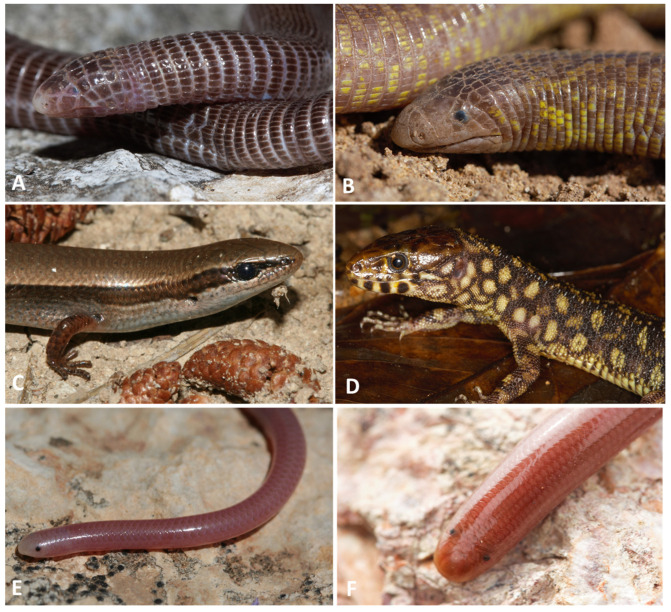
A varying degree of modification of the eyelids is present in several squamata lineages. In worm lizards (Amphisbaenia), such as the Turkish worm lizard (*Blanus strauchi*) (**A**) and the checkerboard worm lizard (*Trogonophis wiemanni*) (**B**), reduced eyes are covered by an ocular scale. (**C**) The Budak’s snake-eyed skink (*Ablepharus budaki*) and other members of its genus have a true spectacle and are the only known reptile species that possess a lacrimal gland. (**D**) In all members of the family Xantusiidae, such as the Costa Rican tropical night lizard (*Lepidophyma reticulatum*), moveable eyelids have been replaced by a true spectacle. Ocular scales that extend well beyond the margins of the globe are present in blind snakes such as the long-nosed worm snake (*Myriopholis macrorhyncha*) (**E**) and the Eurasian blind snake (*Xerotyphlops vermicularis*) (**F**). ©Frank Pasmans.

## Data Availability

Not applicable.
